# G-Protein Subunit Gα_i_ in Mitochondria, MrGPA1, Affects Conidiation, Stress Resistance, and Virulence of Entomopathogenic Fungus *Metarhizium robertsii*

**DOI:** 10.3389/fmicb.2020.01251

**Published:** 2020-06-16

**Authors:** Youmin Tong, Hao Wu, Zhenbang Liu, Zhangxun Wang, Bo Huang

**Affiliations:** ^1^Anhui Provincial Key Laboratory of Microbial Pest Control, Anhui Agricultural University, Hefei, China; ^2^School of Life Sciences, University of Science and Technology of China, Hefei, China; ^3^School of Plant Protection, Anhui Agricultural University, Hefei, China

**Keywords:** *Metarhizium robertsii*, Gα_i_ protein, appressorium, conidiation, virulence

## Abstract

G proteins are critical modulators or transducers in various transmembrane signaling systems. They play key roles in numerous biological processes in fungi, including vegetative growth, development of infection-related structures, asexual conidiation, and virulence. However, functions of G proteins in entomopathogenic fungi remain unclear. Here, we characterized the roles of MrGPA1, a G-protein subunit Gα_i_, in conidiation, stress resistance, and virulence in *Metarhizium robertsii*. MrGPA1 was localized in the mitochondria. *MrGpa1* deletion resulted in a significant reduction (47%) in the conidiation capacity, and reduced expression of several key conidiation-related genes, including *fluG*, *flbD*, *brlA*, *wetA*, *phiA*, and *stuA*. Further, *MrGpa1* disruption resulted in decreased fungal sensitivity to UV irradiation and thermal stress, as determined based on conidial germination of Δ*MrGpa1* and wild-type (WT) strains. Chemical stress analysis indicated that *MrGpa1* contributes to fungal antioxidant capacity and cell wall integrity, but is not involved in tolerance to antifungal drug and osmotic stress. Importantly, insect bioassays involving (topical inoculation and injection) of *Galleria mellonella* larvae revealed decreased virulence of Δ*MrGpa1* strain after cuticle infection. This was accompanied by decreased rates of appressorium formation and reduced expression of several cuticle penetration-related genes. Further assays showed that *MrGpa1* regulated intracellular cyclic AMP (cAMP) levels, but feeding with cAMP could not recover the appressorium formation rate of Δ*MrGpa1.* These observations suggest that *MrGpa1* contributes to the regulation of conidiation, UV irradiation, thermal stress response, antioxidant capacity, and cell wall integrity in *M. robertsii*. This gene is also involved in insect cuticle penetration during infection. These findings raise the possibility of designing powerful strategies for genetic improvement of *M. robertsii* conidiation capacity and virulence for killing pests.

## Introduction

G protein with GTP-hydrolase activity is a type of signaling protein that binds to guanine nucleotides (Robishaw and Berlot, 2004). It participates in signal transduction pathways linking activated cell-surface receptors with intracellular effectors, including adenylate cyclase and phospholipase through a series of signaling cascades involved in the regulation of physiological and biochemical processes (Ortiz-Urquiza and Keyhani, 2015; Chakravorty and Assmann, 2018). In fungi, G protein is associated with sexual and asexual reproduction, virulence, and response to external signal stimuli (Ivey et al., 1996; Liu and Dean, 1997; Guo et al., 2016b).

The heterotrimeric G protein is composed of three subunits α, β, and γ, wherein α subunit binds to GDP, and β and γ subunits form a heterodimer (Birnbaumer, 2007; Lambert, 2008; McIntire, 2009). When the heterotrimeric G protein is stimulated by a G-protein-coupled receptor (GPCR) that senses external signals, GDP is exchanged for GTP, and Gα and Gβγ complexes dissociate (Wedegaertner, 2012). Then, Gα-GTP and Gβγ act on the respective downstream effectors (Barren and Artemyev, 2007). The cycle is reset by the hydrolysis of GTP to GDP, and Gα recombining with Gβγ and GPCR (Gilchrist et al., 1999; Slessareva and Dohlman, 2006).

In mammals, G-protein α (GPA) subunits are divided into four classes, Gα_s_, Gα_i_, Gα_q_, and Gα_12_, based on the amino acid sequence identity (Neer, 1995). Further, the Gα_i_ family is composed of four subfamilies, Gα_i_, Gα_o_, Gα_t_, and Gα_z_. The functions of Gα_i_ family proteins are diverse, and include regulation of adenylyl cyclase, K^+^ and Ca^+^ channels, and cGMP phosphodiesterase activities (Simon et al., 1991). The conserved functional motif of Gα_i_ protein is characterized by possession of *N*-myristoylation and ADP-ribosylation (Buss et al., 1987).

The functions of the GPA subunit have been characterized in some fungi. In *Saccharomyces cerevisiae*, two kinds of Gα proteins have been identified (*Gpa1* and *Gpa2*). *Gpa1* is involved in pheromone regulation (Jahng et al., 1988), while *Gpa2* regulates pseudohyphal development via cyclic AMP (cAMP)-dependent pathways and heat resistance (Kubler et al., 1997). However, these proteins have different functions in other filamentous fungi. For example, *Fusarium oxysporum* f. sp. *cubense* possesses three Gα proteins (Gα-*fga1*, -*fga2*, and -*fga3*), and the deletion of encoding genes leads to phenotypic defects in colony morphology, reduced conidiation, increased heat tolerance, reduced virulence, and decreased intracellular cAMP levels (Jain et al., 2002; Guo et al., 2016a, b). Further, three different Gα proteins control unique signal transduction pathways in *Magnaporthe grisea*, influencing fungal vegetative growth, conidiation, conidium attachment, appressorium formation, mating, and pathogenicity (Liu and Dean, 1997; Zhang et al., 2012). GNA-1 protein is also required for the extension of basal hypha, growth, conidiation, and formation of female reproductive structures in *Neurospora crassa* (Ivey et al., 1996; Yang and Borkovich, 1999). In entomopathogenic fungus, some upstream and downstream genes for *Gpa*, such as GPCRs gene *BbGpcr3*, and regulators of the G protein signaling (RGS) genes *Bbrgs1* and *Mrcag8*, have been only characterized (Fang et al., 2007, 2008; Ying et al., 2013), but the function of *Gpa* in insect pathogenic fungi is poorly understood. Thus, it is necessary to characterize the Gα proteins, and figure out whether the Gα proteins are involving in vegetative growth, conidiation, stress resistance, and virulence in entomopathogenic fungi, such as *Metarhizium robertsii*.

*Metarhizium robertsii*, an important entomopathogenic fungus, has been developed as an environmentally friendly alternative to chemical insecticides (Frazzon et al., 2000; Lord, 2005; Wang and Wang, 2017). Unfortunately, commercialized broad application of *M. robertsii* formulations is limited by the low conidiation rate, failure of conidia germination under high-temperature and UV stress, slow killing speed, and inconsistent field performance (Faria and Wraight, 2007; Fang et al., 2012; Muniz-Paredes et al., 2017). Genetic improvements of this mycoinsecticide require extensive understanding of the molecular mechanisms and *M. robertsii* genes involved in stress tolerance and virulence (Zhang and Feng, 2018).

In the current study, we aimed to investigate the role of Gα proteins in *M. robertsii.* BLASTP search of against the assembled draft genome sequence of *M. robertsii* identified four putative Gα proteins. Among these Gα proteins, MrGPA1 (EFZ00892) shared the highest identity (96.32%) with GNA-1. We then characterized the biological function of MrGPA1 by constructing and analyzing *MrGpa1* gene deletion mutant. We show that *MrGpa1* influences conidiation, stress resistance, virulence, and intracellular cAMP levels in *M. robertsii.*

## Materials and Methods

### Fungal Strains and Culture

In the present study, *M. robertsii* strain ARSEF 23 was the wild-type (WT) strain. All *M. robertsii* strains were inoculated onto potato dextrose agar (PDA, 20% potato, 2% glucose, and 2% agar, w/v), and cultured at 25°C for 10 days. Conidial suspensions were obtained by vortex-mixing in 0.05% (v/v) Tween-80, and filtered through sterile non-woven fabric to remove mycelial debris.

### Sequence Analysis

To construct the phylogenetic tree of GPA proteins and analyze the structural domains of guanine nucleotide-binding site, amino acid sequences of the GPA subunits were downloaded from the National Center for Biotechnology Information^1^, and phylogenetic analysis was performed using MEGAX software^2^.

### Construction of MrGPA1-GFP Fusion Vector and Analysis of Subcellular Localization of MrGPA1

To monitor subcellular localization of *MrGpa1*, *gfp* and *MrGpa1* gene fragments were amplified by polymerase chain reaction (PCR), using *gfp*-F/*gfp*-R, and *gfpMrGpa1*-F/*gfpMrGpa1*-R primers (Supplementary Table S1), high-fidelity Taq DNA polymerase (KOD Plus Neo, Toyobo, Osaka, Japan), and *M. robertsii* genomic DNA as a template. The amplification products were inserted into the *Eco*RI restriction site in pDHt-SK-*bar* vector (kindly provided by Dr. Chengshu Wang; the vector conferred resistance against glufosinate-ammonium) (Fang et al., 2006) containing a strong promoter and terminator to generate vector pDHt-*MrGpa1*-*gfp* for *Agrobacterium tumefaciens* transformation. The corresponding transformants resistant to glufosinate ammonium were obtained, and verified by PCR using the primers *gfp*-F and *gfp*-R (Supplementary Table S1).

The MrGPA1-GFP strain was cultured on sabouraud dextrose agar medium containing yeast extract (SDAY, 4% glucose, 1% peptone, 2% agar, and 1% yeast extract powder, w/v) at 25°C for 2 days. The hyphae were then washed off the plate with sterile water and mixed with 500 nM MitoTracker Red CMXRos (Invitrogen, Shanghai, China), a dye specific to mitochondria. Subcellular localization of MrGPA1 was evaluated using a laser scanning confocal microscopy (LSCM, Zeiss LSM880). Before using laser scanning confocal microscopy, Wolf PSORT software^3^ was used for prediction subcellular localization by analyzing protein sequence of MrGPA1.

### Gene Deletion and Complementation

To disrupt *MrGpa1* gene, the 5′- and 3′-flanking regions of *MrGpa1* were obtained by using *MrGpa1*-5F/*MrGpa1*-5R and *MrGpa1*-3F/*MrGpa1*-3R primers, genomic DNA (using the Plant Genomic DNA Kit; Tiangen, Beijing, China) extracted as a PCR template and high-fidelity Taq DNA polymerase (KOD Plus Neo, Toyobo, Osaka, Japan). The amplification products were then inserted into the pDHt-SK-*bar* vector (containing glufosinate resistance gene) (Fang et al., 2006) digested with the *Sma*I, *Bam*HI, and *Xba*I restriction enzymes to generate vector pDHt-*MrGpa1*-*bar* for *A. tumefaciens* transformation (Fang et al., 2006). Δ*MrGpa1* strains were obtained by selection for glufosinate resistance, and two fragments containing the *MrGpa1*- upstream-*bar*-*MrGpa1*-downstream cassette subsequently verified by PCR and reverse-transcription (RT)-PCR using primer pairs *MrGpa1*-F/*MrGpa1*-R, up*MrGpa1*-F/up*MrGpa1*-R, dn*MrGpa1*-F/dn*MrGpa1*-R, *gpd*-F/*gpd*-R, and *bar*-F/*bar*-R (Supplementary Table S1).

For gene complementation, the entire *MrGpa1* gene and the 1000-bp upstream sequence and 600-bp downstream sequence were inserted into vector pDHt-SK-*ben* (containing benomyl resistance gene) digested with the *Spe*I restriction enzyme for fungal transformation. The 3050-bp fragment was ectopically integrated into Δ*MrGpa1* strain by the same method as that used for gene deletion. Complemented strains (cpΔ*MrGpa1*) were obtained by selection for benomyl resistance, and verified by PCR using primer pairs *MrGpa1*-F/*MrGpa1*-R and *ben*-F/*ben*-R (Supplementary Table S1).

### Phenotype Assays

For phenotype assays, these experiments were performed with three technical and biological replicates per strain (WT, Δ*MrGpa1*, and cpΔ*MrGpa1*).

Fungal conidiation ability was evaluated as previously described (Meng et al., 2017). Briefly, 30 μl of conidial suspension (1 × 10^6^ conidia/ml) was spread on PDA plate (35-mm diameter). After culturing at 25°C for 14 days, the conidia on each plate were collected into 30 mL of 0.05% Tween-80 by vortex-mixing, and conidial density was determined using a hemocytometer and converted to the number of conidia per square centimeter of colony.

To evaluate the fungal vegetative growth, 1 μl of WT, Δ*MrGpa1*, and cpΔ*MrGpa1* conidial suspensions (1 × 10^7^ conidia/ml) was spotted on PDA and 1/4 SDAY (1/4 dilution of SDAY) media, and incubated in the dark at 25°C for 10 days. Colony diameters were then measured.

For conidial germination assay, 10 μl of conidial suspension (5 × 10^6^ conidia/ml) were spread on PDA medium. The conidial germination was observed by microscope (Olympus BX 51, Tokyo, Japan) at 2, 4, 6, 8, 10, 12, 14, 16, 18, 20, 22, 24 h after incubated at 25°C. Conidia are considered to be germinated when the length of the germ tube reaches or longer than the length of the conidia (Wang et al., 2014). Three hundred conidia were counted at least per plate and the germination rates were calculated by comparing the number of germinated conidia with the 300 counted conidia, and the median germinate time (GT_50_) was calculated using the SPSS software.

For heat stress tolerance assays, 1 ml of conidial suspensions (5 × 10^6^ conidia/ml) of WT, Δ*MrGpa1*, and cpΔ*MrGpa1* strains were placed in 1.5-ml Eppendorf tubes, and then incubated in a water bath at 42 or 28°C (as control) for 1 h. Then 10 μl of the suspension were spread on PDA medium, incubated at 25°C. Conidial germination was observed under a microscope (Olympus BX 51, Tokyo, Japan) after 16 and 24 h. Three hundred conidia were counted at least by per plate and the relative germination rates were calculated by comparing the number of germinated conidia with had not been heat stressed (Wang et al., 2019).

To determine fungal tolerance to ultraviolet B (UV-B) light, 10 μl of conidial suspensions (5 × 10^6^ conidia/ml) of WT, Δ*MrGpa1*, and cpΔ*MrGpa1* was taken to PDA medium. The plates were then exposed to UV-B irradiation (312-nm wavelength at 100 μJ cm^–2^) using HL-2000 Hybrilinker (UVP, CA, United States) (Yao et al., 2010) or exposed to sunlight (as control). Relative UV-B tolerance was assessed and calculated by aforementioned methods to assess relative germination rate of tolerance to UV-B.

To examine the fungal tolerance to chemical stress, 1 μl conidial suspensions (1 × 10^7^ conidia/ml) of WT, Δ*MrGpa1*, and cpΔ*MrGpa1* strains were spotted onto PDA medium containing carbendazim (2 μg/ml), NaCl (0.5 M), H_2_O_2_ (2 mM), or Congo red (2 μg/ml), and incubated in the dark for 10 days at 25°C. Colony diameter was then measured and the relative inhibition rate was calculated (Ying and Feng, 2011; Wang et al., 2017).

To assess the effects of *MrGpa1* disruption on virulence, bioassays with *Galleria mellonella* larvae (RuiQing Bait, Shanghai, China) were performed as described previously (Zhou et al., 2018). The larvae were immersed in conidial suspension (1 × 10^6^ conidia/ml) for 90 s or injected (into the hemocoel) with 10 μl of conidial suspensions (1 × 10^5^ conidia/ml) and incubated at 25°C, each treatment was performed in triplicate, with 18 larvae in each group. The experiment was repeated three times. Larva mortality was evaluated every 24 h, and the median lethal time (LT_50_) was calculated using the SPSS software.

The appressorium formation assay was performed as described previously (Gao et al., 2013). Briefly, to test the appressorium formation on a hydrophobic surface, 1 ml of conidial suspension (1 × 10^6^ conidia/ml) in MMGly (minimal medium amended with 1% glycerol) was spread on a sterile plastic Petri dishes (3.5-cm diameter), followed by 24 and 48 h incubation at 25°C. At least 300 conidia of each strain were evaluated microscopically, and the induction rates of appressorium formation were quantified by observing different microscopic fields (inverted microscope, Olympus IX 71, Tokyo, Japan).

### Quantitative RT-PCR (RT-qPCR)

To analyze the expression of conidiation-related genes, 200 μl of conidial suspensions (1 × 10^7^ conidia/ml) of WT, Δ*MrGpa1*, and cpΔ*MrGpa1* strains were plated on PDA medium, and cultured in the dark at 25°C for 2.5 days. The samples were collected and milled in liquid nitrogen to extract total RNA. To analyze the expression of virulence genes related to cuticle infection, *G. mellonella* larvae were dipped in conidial suspensions (5 × 10^7^ conidia/ml) of WT, Δ*MrGpa1*, and cpΔ*MrGpa1* strains for 1.5 min, transferred to 25°C for 48 h, and then placed in liquid nitrogen for total RNA extraction. Total RNA was extracted by using Trizol reagent (Invitrogen, Foster City, CA, United States). cDNA was obtained by using the PrimeScript^TM^ RT reagent kit with gDNA Eraser (TaKaRa, Dalian, China), and used as a template for RT-qPCR. The gene expression analysis was performed by using the CFBR96^TM^ Real-Time PCR System (Bio-Rad, Hercules, CA, United States) and SYBR^®^ PremixEx TaqTM II (TaKaRa). Three biological repeats of each treatment were analyzed. The qPCR primers are listed in Supplementary Table S2. The expression of the *gpd* gene (MAA_07675, encoding glyceraldehyde 3-phosphate dehydrogenase) was used as an internal control (Fang and Bidochka, 2006). The relative gene expression was calculated by using the 2^–ΔΔCt^ method (Livak and Schmittgen, 2001).

### cAMP Assay

To measure the intracellular cAMP levels of WT, Δ*MrGpa1*, and cpΔ*MrGpa1* strains, the method was performed previously (Liu et al., 2012). The cAMP levels were measured by high-performance liquid chromatography (HPLC) analysis as described previously (Liu et al., 2016).

To test whether exogenous cAMP could recover appressorium formation rate of Δ*MrGpa1*, the conidial suspensions (1 × 10^6^ conidia/ml) were amended with the final concentration of 5 mM cAMP and left for appressorium formation on a hydrophobic surface as mentioned above.

### Statistical Analysis

All data are presented using GraphPad Prism version 6.0. Data are expressed as the mean ± standard error (SE) of the mean, from three biological replicates. Statistical analysis was performed by one-way analysis of variance (ANOVA). For multiple comparisons, Tukey’s multiple comparison test was used to analyze statistical the significance. *p* < 0.05 was considered to be significant, and *p* < 0.01 was considered to be extremely significant.

## Results

### Sequence Characteristics of MrGPA1 From *M. robertsii*

We chose the sequences of GNA-1 proteins of the model fungus *N. crassa* as reference sequences to retrieve their *M. robertsii* orthologs. We thus identified four genes encoding GPA subunit [GenBank accession numbers EFZ00892.1, EFY98464.1, EFY99066.2, and EFZ00060.2, named *MrGpa1*, *MrGpa2*, *MrGpa3*, and *MrGpa4* (i.e., the *M. robertsii Gpa1*, *Gpa2*, *Gpa3*, and *Gpa4* genes), respectively] in the genome of *M. robertsii* ARSEF 23.

Further bioinformatics analysis indicated that *MrGpa1* (MAA-03488) is a single copy gene encoding GPA subunit (353-aa protein) in *M. robertsii*. A BLASTP search of MrGPA1 homologs in NCBI revealed that the protein shares 100% amino acid similarity with GPA from *Metarhizium acridum* (XP_007811324), *Metarhizium anisopliae* (KFG82129), *Metarhizium brunneum* (XP_014542423), *and Metarhizium rileyi* (OAA44756). Phylogenetic tree of GPA proteins from *Metarhizium* spp., and related fungal species was constructed with *S. cerevisiae* as an outgroup (Supplementary Figure S1A). All GPA proteins from the genus *Metarhizium* formed an independent branch (100% support value). These *Metarhizium* GPA proteins are closely related to GPA proteins from *Pochonia chlamydosporia* (99.72%), *Moelleriella libera* (99.43%), and *Purpureocillium lilacinum* (99.15%).

A conserved domain database^4^ search demonstrated that MrGPA1 contains a highly conserved guanine nucleotide-binding site (34-347-aa protein), which is the key identification domain of the GPA subunit (Supplementary Figure S1B). Furthermore, homologous alignment revealed the presence of *N*-myristoylation and ADP-ribosylation sites (two conserved positions in the GPA_i_ subunit) in the conserved functional motif of MrGPA1. Hence, MrGPA1 is a member of the Gα_i_ family.

### MrGPA1 Is a Mitochondria Protein

**FIGURE 1:**
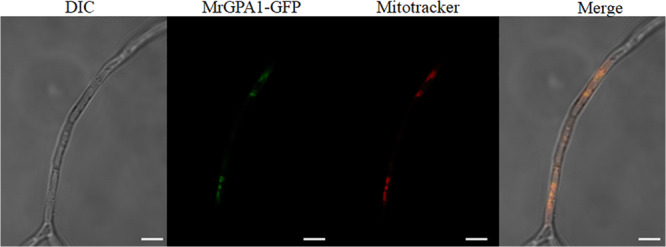
MrGPA1 is a mitochondria protein. LSCM images (scale bars: 10 μm) of the cellular location of MrGPA1, showing that MrGPA1 is located in the mitochondria, with the GFP fluorescence signal apparent in hyphae.

To investigate the subcellular localization of MrGPA1, the Wolf PSORT software was first used. The analysis predicted that the protein is localized in the mitochondria. To verify this prediction, we generated *MrGpa1*-*gfp* strain (Supplementary Figures S2A,B). As shown in LSCM images in Figure 1, the mitochondria in hypha is stained with a mitochondrial dye (red), in a punctate patterns, while punctate green fluorescence was also observed in vegetative hyphae. Red and green fluorescence was detected and overlapped (Figure 1), suggesting that MrGPA1 is a mitochondria protein.

### Construction of *MrGpa1* Knockout and Complementation

The result indicated the presence of a 1, 222-bp fragment corresponding to the partial *MrGpa1* gene sequence in the WT and cpΔ*MrGpa1* stains, but not in the Δ*MrGpa1* strain. In addition, a partial 806-bp *bar* gene fragment was detected in Δ*MrGpa1* and cpΔ*MrGpa1* strains, and a partial 785-bp *ben* gene fragment was detected in the cpΔ*MrGpa1* strain. Furthermore, PCR analysis indicated the presence of a fragment containing upstream sequence of *MrGpa1* and a partial *bar* gene (2689 bp) and a fragment containing downstream sequence of *MrGpa1* and a partial *bar* gene (1975 bp), and detected by using the primer sets up *MrGpa1*-F/up*MrGpa1*-R and dn*MrGpa1*-F/dn*MrGpa1*-R, respectively, in the Δ*MrGpa1* strain. Finally, RT-PCR analysis verified the loss or regain of the *MrGpa1* gene expression in Δ*MrGpa1* and cpΔ*MrGpa1* strains, accordingly. These observations indicated a successful construction of the *MrGpa1* knockout and complementation strains (Supplementary Figure S2).

### *MrGpa1* Contributes to Fungal Conidiation but Is Not Involved in Vegetative Growth

**FIGURE 2:**
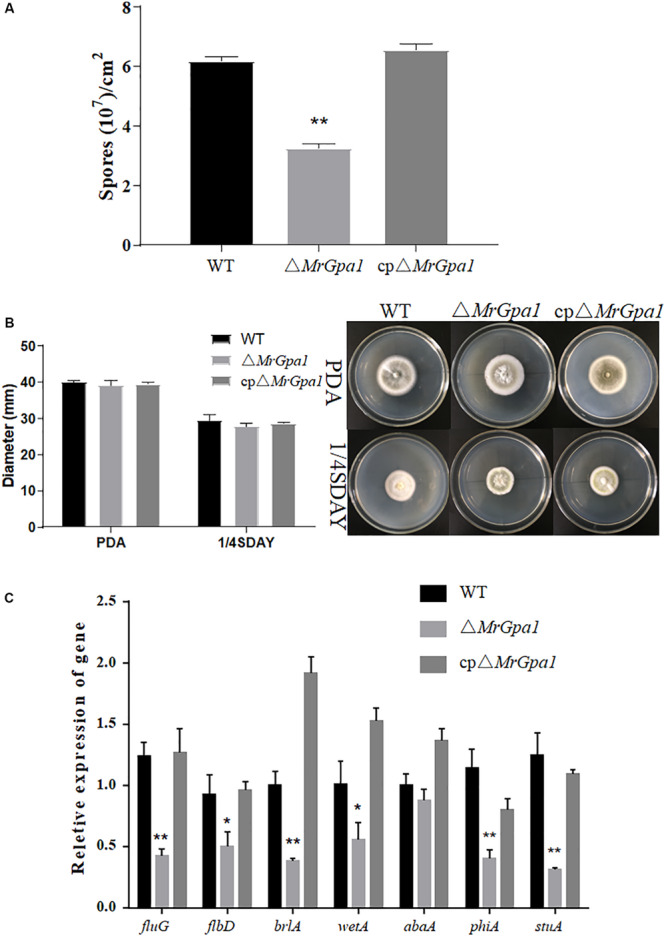
*MrGpa1* contributes to fungal conidiation but is not involved in vegetative growth. **(A)** Conidial yields of three fungal strains after 14-days, growth on PDA at 25°C. **(B)** Colony diameters and phenotype of three fungal strains on PDA and 1/4SDAY media after 10-days, growth at 25°C. **(C)** RT-qPCR analysis of the relative expression of conidiation-related genes in 2.5-day-old PDA cultures of three fungal strains. *p < 0.05, **p < 0.01.

To examine the effect of *MrGpa1* on the growth and development of *M. robertsii*, we evaluated mycelial growth and conidial yield of WT and mutant strains on PDA and 1/4 SDAY medium, respectively. The 14-day-old colonies of WT, Δ*MrGpa1*, and cpΔ*MrGpa1* strains formed 6.18 × 10^7^, 3.28 × 10^7^, and 6.55 × 10^7^ conidia/cm^–2^, respectively (Figure 2A). The loss of *MrGpa1* caused a significant, 47% reduction in conidiation, but little difference in the growth rate of WT, Δ*MrGpa1*, and cpΔ*MrGpa1* strains on PDA and 1/4 SDAY media was apparent (Figures 2A,B). We also examined the expression of genes involved in conidiation in *M. robertsii* by RT-qPCR. The expression of *fluG*, *flbD*, *brlA*, *wetA*, *phiA*, and *stuA* genes in the *MrGpa1* strain was significantly reduced compared with that in the WT and cpΔ*MrGpa1* strains (Figure 2C). Collectively, these observations indicate that while MrGPA1 plays an important role in the conidiation of *M. robertsii*, it is not involved in vegetative growth.

### *MrGpa1* Is Important for Heat and UV Stresses Tolerance, and Is Involved in Antioxidant Capacity and Cell Wall Integrity of *M. robertsii*

**FIGURE 3:**
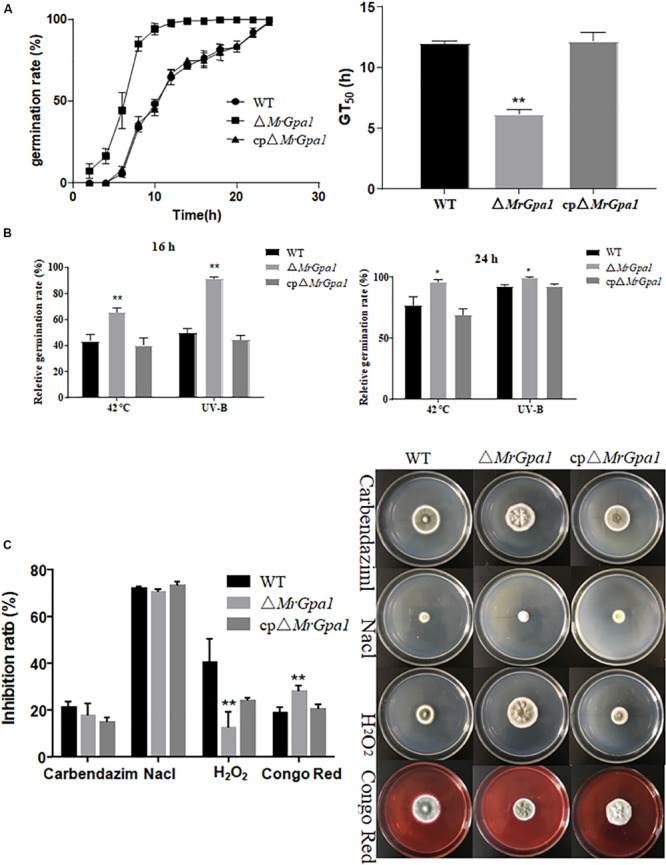
*MrGpa1* is required for fungal stress response. **(A)** Germination rate and GT_50_ values for three fungal strains after growth on PDA. **(B)** Relative germination rate of three fungal strains after exposure to heat stress and UV-B treatment. **(C)** Inhibition ratio and colony phenotype values for three fungal strains, on PDA medium containing Carbendazim (2 μg/ml), NaCl (0.5 M), H_2_O_2_ (10 mM), or Congo red (2 mg/ml). *p < 0.05, **p < 0.01.

The GT_50_ values for WT, Δ*MrGpa1*, and cpΔ*MrGpa1* were 11.99 h, 6.16 h (*p* < 0.01, compared with WT strain), and 12.16 h, respectively (Figure 3A). Conidial germination speed of Δ*MrGpa1* strain on PDA medium was significantly faster than that of WT and cpΔ*MrGpa1* strains.

To investigate the effects of *MrGpa1* deletion on UV irradiation and thermal stress, the relative germination rate of conidia exposed to these stresses was determined 16 or 24 h after stress exposure. We found that the sensitivity of Δ*MrGpa1* strain to 42°C heat stress was reduced. For example, compared with the WT, Δ*MrGpa1* germination rates at 16 and 24 h increased by 51% (*p* < 0.01) and 24% (*p* < 0.05), respectively (Figure 3B). Similar results were obtained for conidial tolerance of UV irradiation; compared with the WT, Δ*MrGpa1* germination rates at 16 h increased by 83% (*p* < 0.01), but only by 8% (*p* < 0.05) at 24 h (Figure 3B). Hence, it appears that MrGPA1 plays an important role in conidial tolerance of both UV irradiation and thermal stress.

To evaluate the role of *MrGpa1* in fungal growth under different chemical stress conditions, we investigated the mycelial growth of the WT and mutant strains on PDA containing carbendazim, NaCl, H_2_O_2_, or Congo red. The antioxidant capacity and cell wall integrity of Δ*MrGpa1* strain were significantly different than those of the WT and cpΔ*MrGpa1* strains. For instance, compared with the WT, the relative inhibition of Δ*MrGpa1* growth was decreased by 68.6% (*p* < 0.01) on PDA containing H_2_O_2_, while the sensitivity to Congo red was increased by 47.4% (*p* < 0.01). However, the relative inhibition of Δ*MrGpa1* growth in the presence of carbendazim and NaCl was not markedly different from that of the control strains (Figure 3C). These observations indicate that *MrGpa1* contributes to fungal antioxidant capacity and cell wall integrity, but is not involved in antifungal ability and osmotic stress.

### *MrGpa1* Plays an Important Role in Insect Cuticle Penetration via Appressorium Formation

**FIGURE 4:**
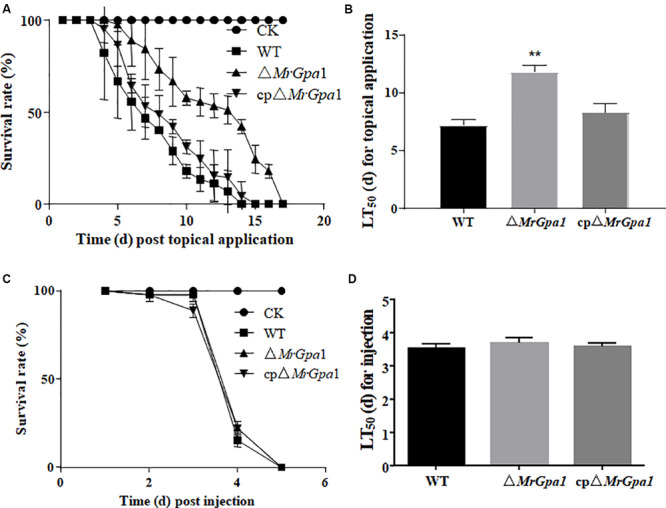
*MrGpa1* deletion impacts fungal virulence. **(A)** Survival of *G.mellonella* after topical application of conidial suspensions from three fungal strains. The control insects were treated with sterile water. **(B)** LT_50_ (days) of three fungal strains after topical inoculation of larvae. **(C)** Survival of *G. mellonella* after injection of conidial suspension of three fungal strains. The control insects were treated with sterile water. **(D)** LT_50_ (days) of three fungal strains after injection. **p < 0.01.

We next used *G. mellonella* bioassays to assess the consequences of *MrGpa1* deletion on fungal virulence. In topical infection bioassays, the mean lethal times to death (LT_50_) in insects infected with Δ*MrGpa1*, WT, and cpΔ*MrGpa1* strains were 7.2 ± 0.45, 11.8 ± 0.54, and 8.3 ± 0.71 days, respectively, with a significant (*p* < 0.05) attenuation of virulence in *G. mellonella* (Figures 4A,B). The treatment with Δ*MrGpa1* also resulted in an increased survival rate of the larvae compared with the WT and cpΔ*MrGpa1* strains treatment. By contrast, in the injection bioassays, we did not observe any differences in LT_50_ values between larvae infected with Δ*MrGpa1* (3.69 ± 0.15 days), and WT (3.53 ± 0.14 days) or cpΔ*MrGpa1* strains (3.57 ± 0.12 days) (Figures 4C,D).

**FIGURE 5:**
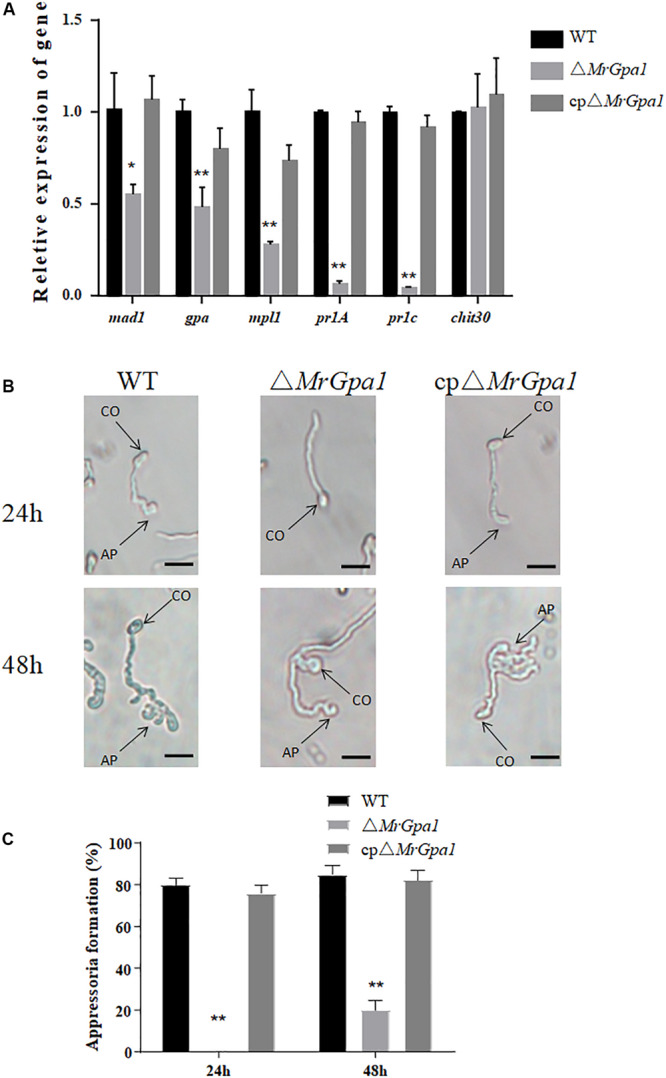
*MrGpa1* deletion affects appressorium formation. **(A)** RT-qPCR analysis for the relative expression of virulence-associated genes of three fungal strains *in vivo* (24 h after inoculation of *G. mellonella* larvae). **(B)** Microscopic analysis and percentage appressorium formation by fungal strains induced on a plastic hydrophobic surface. Scale: 10 μm. **(C)** Percentage of appressorium formation *in vitro*. *p < 0.05, **p < 0.01. CO, conidium, AP, appressorium.

We then determined the expression of insect virulence-related genes during cuticle penetration by RT-qPCR. Indeed, the expression of several genes involved in the adhesion (*mad1*, 45% expression in Δ*MrGpa1* strain compared with the WT strain), appressorium formation (*mpl1*, 72%, and *gpa*, 52%), and cuticle penetration (*pr1A*, 93%, and *pr1C*, 96%) was significantly decreased in the Δ*MrGpa1* strain compared with their expression in the WT strains (Figure 5A).

To determine the mechanism of the virulence defect of Δ*MrGpa1* strain, we then assayed appressorium formation on a hydrophobic surface. We observed that the loss of *MrGpa1* impaired appressorium differentiation, compared with the control strains. Specifically, 24 h after induction, Δ*MrGpa1* strain did not form appressoria, while the appressorium formation rate of the WT strain was 80% (Figure 5B). Further, 48 h after induction, the appressorium formation rate of Δ*MrGpa1* was only approximately 20% and were significantly reduced (by 76.5%) compared with WT strains (Figure 5C). Therefore, *MrGpa1* plays an important role in cuticle penetration by impacting appressorium formation.

### *MrGpa1* Regulates Intracellular cAMP Levels, but Further Feeding With cAMP Cannot Recover the Appressorium Formation Rate of Δ*MrGpa1*

**FIGURE 6:**
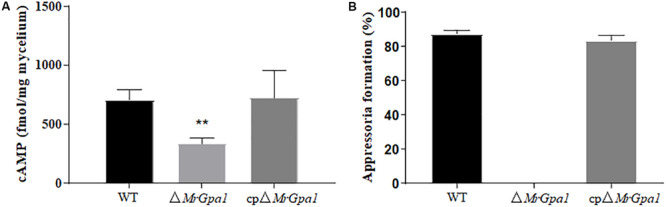
**(A)** Quantification of intracellular cAMP in the hyphal stage. **(B)** After 24 h incubation, the percentage of appressorium formation by adding exogenous 5 mM cAMP. **p < 0.01.

To determine whether *MrGpa1* regulates cAMP levels in *M. robertsii*, the intracellular cAMP levels were measured in the hyphal stage. We found that the intracellular cAMP levels of Δ*MrGpa1* were significant decreased by 52.8% (*p* < 0.01), compared with WT strains (Figure 6A). The result indicated that *MrGpa1* is a positive regulator in intracellular cAMP levels of *M. robertsii*. However, the appressorium formation rate of Δ*MrGpa1* cannot recover with the addition of 5 mM exogenous cAMP (Figure 6B).

## Discussion

G-proteins are key components of various signal transduction pathways and play show important biological roles in the control of cell proliferation, behavior, and development in higher organisms (Ivey et al., 1996; Regenfelder et al., 1997; Harashima and Heitman, 2005). In the current study, we described the identification of *MrGpa1* gene encoding the Gα_i_ subunit in *M. robertsii*, and showed that this Gα_i_ protein plays an important role in conidiation, stress resistance, and virulence in the host fungus.

In filamentous fungi, genes encoding three GPA subunits were reported in *N. crassa*, *F. oxysporum* f. sp. *cubense*, *Aspergillus nidulans*, and *M. grisea* (Ivey et al., 1996; Hicks et al., 1997; Liu and Dean, 1997; Guo et al., 2016b). However, while genes of four G protein α subunits were identified in *M. robertsii*, two of the open reading frames (ORFs) are highly similar (*MrGpa2* and *MrGpa4*). Further, the existence of Gα_i_ has been demonstrated in filamentous fungi including the *M. robertsii* in this study, but not in the yeasts *S. cerevisiae* or *Schizosaccharomyces pombe* (Kubler et al., 1997; Kallal and Fishel, 2000).

Previously, to be activated by GPCR that sense external signals in plasma membrane (PM), G protein was considered to locate at the PM (Marrari et al., 2007). However, recent studies revealed G-protein localization beyond the PM, e.g., in the mitochondrion, endoplasmic reticulum, and Golgi apparatus (Michaelson et al., 2002; Nair et al., 2017). Wolf PSORT prediction in the current study indicated a possible location of MrGPA1 in the mitochondrion. Indeed, by using a fluorescent protein fusion, we showed here that MrGPA1 is located in the mitochondria, which is consistent with location of Gα_i_ in HEK293T cells (Lyssand and Bajjalieh, 2007). It have been reported that the roles of mitochondria in fungi is involved in the aging, conidiation, tolerance to adverse stresses and pathogenesis (Lorin et al., 2006; Kretschmer et al., 2012; Kitagaki and Takagi, 2014; Khan et al., 2015). For example, previous report in *Magnaporthe oryzae* shows that mitochondrial fission protein MoFis1 mediates conidiation and is required for full virulence (Khan et al., 2015). Moreover, Kretschmer et al. (2012) found that defects in mitochondrial influence virulence from *Ustilago maydis*. Therefore, the biological roles of MrGPA1 localized to mitochondria are further investigated in the present study.

According to multiple studies, GPA subunits play a role in the conidial yield in some fungi. For example, the conidiation of *magB* deletion mutant of *M. grisea* and Δ*fga1* mutant of *F. oxysporum* f. sp. *cubense* is impaired (Liu and Dean, 1997; Guo et al., 2016b). A similar phenomenon was also observed in the current study. Because a conserved conidiation regulatory pathway in *M. robertsii* contains Mr-BrlA, Mr-AbaA, and Mr-WetA (Zeng et al., 2017), these key genes were downregulated in the *MrGpa1* deletion mutant too. All these indicated that the *MrGpa1* gene is involved in the conidiation of *M. robertsii* by regulating the expression of conidiation-related genes.

We also observed that Δ*MrGpa1* strain is more tolerant to UV irradiation and thermal stress than the WT and cpΔ*MrGpa1* strains. This observation was consistent with findings for *F. oxysporum* f. sp. *cubense* and *N. crassa* (Yang and Borkovich, 1999; Guo et al., 2016b). According to the two cited studies, intracellular cAMP levels are reduced in the Gα_i_ deletion mutants, suggesting that the cAMP pathway might be involved in the response to thermal stress and tolerance of UV irradiation in some fungi.

In the *M. robertsii*, *MrGpa1* deletion resulted in a marked attenuation of virulence in the *G. mellonella* model, with an increased LT_50_ value (by 63.9%) compared with that of the WT strain. Further analysis indicated that the reduced virulence is associated with an impaired appressorium differentiation in the mutant. This was similar to the effect of *magB* deletion in *M. grisea*, namely, blocked appressorium formation in a deletion strain (Liu and Dean, 1997). The virulence of plant pathogenic fungi is regulated by multiple pathways, such as the mitogen activated protein kinase (MAPK) cascades and the cAMP-PKA pathway (Dean, 1997). Further, the Gα_i_ family contributes to pathogenicity in many plant pathogenic fungi. For example, the pathogenicity of the deletion strains *M. grisea ΔmagB*, *Botryils cinerea Δbcg1*, and *F. oxysporum* f. sp. *cubense Δfga1* is markedly reduced (Liu and Dean, 1997; Gronover et al., 2001; Guo et al., 2016b). Moreover, MAPK cascades and cAMP-PKA pathway are also involved in virulence of entomopathogenic fungus (Fang et al., 2009; Jin et al., 2012; Chen et al., 2016). In this study, the decrease of the intracellular cAMP levels in Δ*MrGpa1* strains demonstrates that *MrGpa1* involved in cAMP-PKA pathway. However, feeding with cAMP cannot recover the appressorium formation rate of Δ*MrGpa1*. In contrast to *magB* deletion in *M. grisea*, the appressorium differentiation is dependent on cAMP (Liu and Dean, 1997). We speculated that *MrGpa1* located in mitochondria and *magB* are different G proteins, and *MrGpa1* is involved in not only cAMP-PKA pathway, but also other regulatory pathways affecting on appressorium differentiation, such as MAKP cascades or RGS pathway.

## Conclusion

MrGPA1 is located in the mitochondria of *M. robertsii* cell and is a member of Gα_i_ family. MrGPA1 controls unique signal transduction pathways, and thus plays important role in conidiation, stress resistance, virulence, and intracellular cAMP levels in that fungus. These findings raise the possibility of designing powerful strategies for genetic improvement of *M. robertsii* conidiation capacity and virulence for killing pests.

## Data Availability Statement

All datasets generated for this study are included in the article/Supplementary Material.

## Author Contributions

BH and ZW conceived and designed the study. YT and HW wrote the manuscript, conducted the experiments, and analyzed the data. ZL did a part of the experiments. BH edited the manuscript and supervised the project. All authors read and approved the manuscript.

## Conflict of Interest

The authors declare that the research was conducted in the absence of any commercial or financial relationships that could be construed as a potential conflict of interest.

## Footnotes

1 https://www.ncbi.nlm.nih.gov/

2 https://www.megasoftware.net/

3 https://wolfpsort.hgc.jp

4 http://www.ebi.ac.uk/interpro/
