# Supplementary material for: G-Protein Subunit Gαi in Mitochondria, MrGPA1, Affects Conidiation, Stress Resistance, and Virulence of Entomopathogenic Fungus Metarhizium robertsii
Source: Front Microbiol. 2020 Jun 16;11:1251. doi: 10.3389/fmicb.2020.01251 (PMC7309505; doi:10.3389/fmicb.2020.01251)
Supplement: Supplementary file 1 [file Data_Sheet_1.doc]

Supplementary Material for *Frontiers in Microbiology*

**Title:** G-protein subunit Gα_i_ in mitochondria, MrGPA1, affects conidiation, stress resistance, and virulence of entomopathogenic fungus *Metarhizium robertsii*

**Authors:** YouMinTong^1, †^·Hao Wu^1, †^·ZhenBang Liu^3^· ZhangXun Wang^1,2^·Bo Huang^1,*^

**Affiliation:** ^1^ Anhui Provincial Key Laboratory of Microbial Pest Control, Anhui Agricultural University, Hefei 230036, China

**Affiliation:** ^2^School of Plant Protection, Anhui Agricultural University, Hefei 230036, China

**Affiliation:** ^3^School of Life Sciences, University of Science and Technology of China, Hefei 230022, China

† These authors contribute equally to this study.

^*^Corresponding authors.

Anhui Provincial Key Laboratory of Microbial Pest Control, Anhui Agricultural University, Hefei 230036, China.

Tel./ Fax: +86-551- 65786211.

E-mail address: bhuang@ahau.edu.cn (Bo Huang)

^#^These authors contributed equally to this study and share first authorship


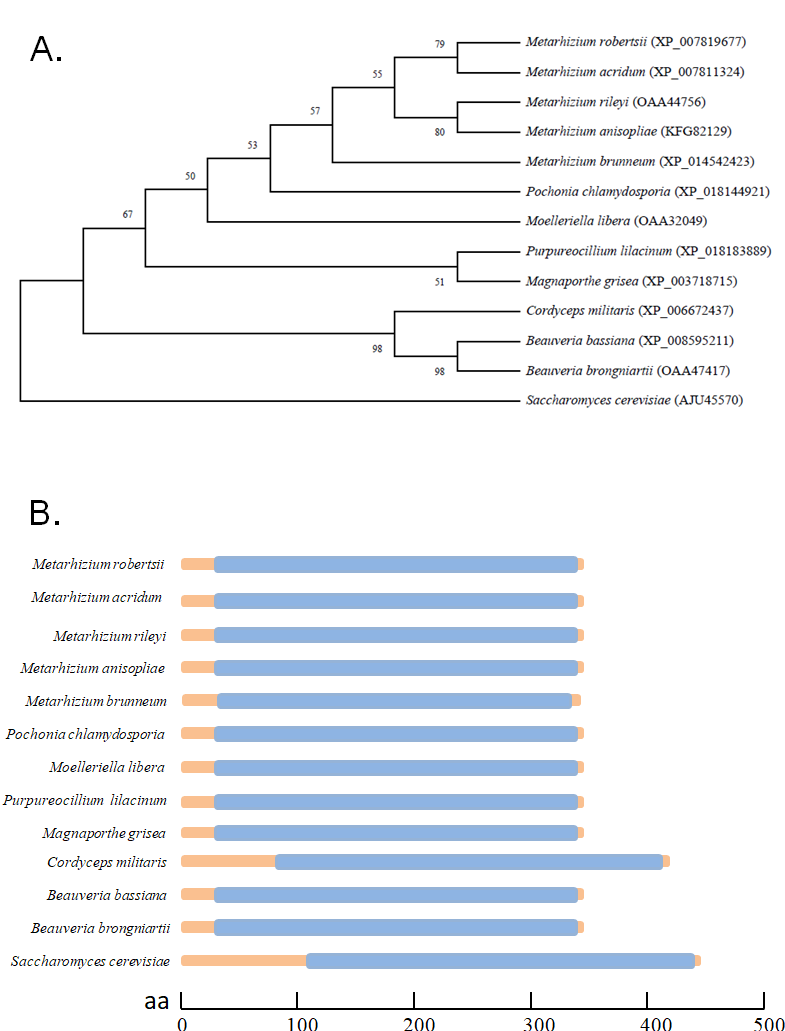


**FIGURE S1 Phylogenetic and conserved domain analysis of MrGPA1.**

1. Phylogenetic analysis of MrGPA1 and its orthologs from different fungi. The National Center for Biotechnology Information accession numbers of MrGPA1 and related proteins are given in brackets following each fungal name.
2. Conserved domain analysis of MrGPA1 and its orthologues from different fungi. The guanine nucleotide-binding domain of the G-protein α subunit is shown in blue. aa, amino acid.

**
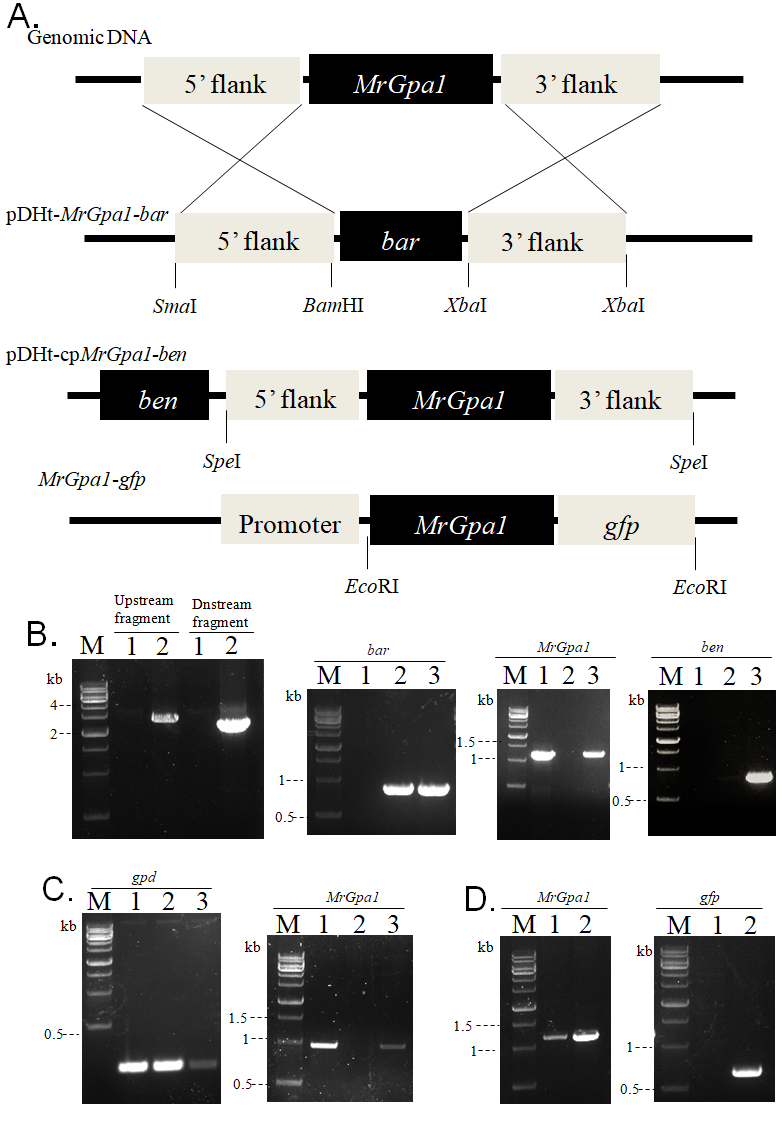
**

**FIGURE S2. Gene deletion , complementation and construction of GFP fusion**

**vector of *MrGpa1* in *M. robertsii*.**

1. Schematic diagram for the *MrGpa1* gene disruption and GFP fusion strains obtain by the homologous recombination method.

The 1222bp fragment of the *MrGpa1* was replaced by the 806-bp of the *bar* cassette through homologous recombination between the overlapping regions of the 5’ and 3’ fragments in *MrGpa1*and pDHT-*bar*-*MrGpa1* (recombinant vector), respectively.

The 1222-bp of fragment of the *MrGpa1* and 720-bp of fragment of *gfp* gene were insert into the pDHt-SK-*bar* vector (containing a strong promoter and terminator).

1. PCR analysis of *MrGpa1* deletion and complementation. The upstream fragment, downstream fragment, *bar*, *MrGpa1*, and *ben* were amplified with the primer sets up*MrGpa1*-F/up*MrGpa1*-R, dn*MrGpa1*-F/dn*MrGpa1*-R, *bar*-F/*bar*-R, *MrGpa1*-F/*MrGpa1*-R, and *ben*-F/*ben*-R, respectively (see Table S1). M: Marker; 1: WT (wild-type strain), 2: *∆MrGpa1*strain, 3: cp*∆MrGpa1* (complemented strain). PCR analysis of *MrGpa1*-*gfp* fusion vector.
2. RT-PCR analysis of *MrGpa1* deletion. The *gpd* and *MrGpa1*were amplified with the primer sets *gpd*-F/*gpd*-R and *MrGpa1*-F/*MrGpa1*-R. M: Marker; 1: WT (wild-type strain), 2: *∆MrGpa1*strain, 3: cp*∆MrGpa1* (complemented strain).
3. The *MrGpa1* and *gfp* were amplified with the primer sets *MrGpa1*-F/*MrGpa1*-R and *gfp*-F/*gfp*-R. M: Marker; 1: WT (wild-type strain), 2: *MrGpa1*-*gfp* strain.

**Table S1 Primers of deletion, complementation and construction of the GFP fusion vector**

| **Gene** | **Primer Name** | **Sequence(5’-3’)** | **Note** |
| --- | --- | --- | --- |
| **Gene deletion** |  |  |  |
| *MrGpa1* | *MrGpa1*-5F | TCCCCCGGGGGCGATGGTGTTAGTTG | PCR idenification of deletion transformants |
|  | *MrGpa1*-5R | CGCGGATCCAGAGGCAGCAGTTGGT |
|  | *MrGpa1*-3F | GCTCTAGACTCAATAACGACCACCCAG |
|  | *MrGpa1*-3R | GCTCTAGAAACGAACTTCCGAGACCAT |  |
|  | up*MrGpa1*-F | TCCTTGTTCTGGTTTCGTTTCA |  |
|  | up*MrGpa1*-R  dn*MrGpa1*-F  dn*MrGpa1*-R  *MrGpa1*-F | GAAGTCCAGCTGCCAGAAACC  TCGTCAACCACTACATCGAGAC  AAACTCCAGAAGGAATCACCGA  GATGCTATTGCTCGGTATGT |  |
|  | *MrGpa1*-R | GTTCTCTTGGATGATGATGT |  |
|  | cp*MrGpa1*-F | GGACTAGTGCAAAGCACAATCAACATC | PCR idenification of complemencation transformants |
|  | cp*MrGpa1*-R | GGACTAGTTCCCAACTCTCTCACCACT |
|  | *MrGpa1*-qpcrF | ATGAGCACAGAGGAGAAGGAGG | RT-PCR analysis |
|  | *MrGpa1*-qpcrR | TGGCGCAGGTGAAATGAGTATA |  |
| *bar* | *bar*-F  *bar*-R | ATTTTGGTTTAGTCGTCCAGGCG  AGCTGCCAGAAACCCACGTCATG | Genomic PCR analysis |
| *ben* | *ben*-F | GATGGCTACCTACTCCGTCGTG |  |
|  | *ben*-R | TCTCGTCCATACCCTCACCAGT |  |
| *gfp* | *gfp*-F | ACGGACGCAGAGAGAAGGG |  |
|  | *gfp*-R | TGGTGGGGAGAGCAGGAAA |  |
| *gpd* | *gpd*-F | GACTGCCCGCATTGAGAAG | Genomic PCR and |
|  | *gpd*-R | AGATGGAGGAGTTGGTGTTG | RT-PCR analysis |

**Table S2 Gene expression analysis**

| **Gene** | **Accession number** | **Annotation** | **Sequence (5’-3’)** |
| --- | --- | --- | --- |
| **Involved in conidiation** | |  |  |
| *fluG* | MAA-00122 | Protein fluG | TGCGGGTTGAATACGG/CTCCACCTCTTTCTCCTTGA |
| *flbD* | MAA-03655 | Conidiophore development protein | AACGATGGGCTGAGATTG/GGTGATTGAGTTTCGGATG |
| *brlA* | MAA-10599 | C_2_H_2_ conidiation transcription factor BrlA | CAACAGCAGGAATCGC/GCTTATCGGCTGACTTTG |
| *wetA* | MAA-02845 | Conidial maturation factor WetA | CGACGAAATAGGAAAGCA/TGAAGTGGAGGAGATACGG |
| *abaA* | MAA-00694 | Conidiation transcription factor AbaA | AAACCACTATTCCTGCTCC/AGCCTGCCTGTTACGATA |
| *phiA* | MAA_06808 | Secretion pathway protein Sls2/Rcy1 | ACGGAGGCGGTTTGAAGA/AATGTCGTCGCTTGTGGC |
| *stuA* | MAA-02988 | APSES transcription factor | GCAAGGCACCAACCCACT/TGCTGCTCCGTAGGCTGA |
| **Involved in virulence** | |  |  |
| *mad1* | MAA-03775 | Adhesion protein 1 insect adhesin | CAACACCTTCGTGGACC/CGTGGGCGTAAATGCA |
| *gpa* | MAA-02162 | Glycerol-3-phosphate acyltransferase | GCTCCTACCCTCTATCACT/GCCAGATGCCAATGTAC |
| *mpl1* | MAA-08819 | Perilipin homolog MPL1 | AACAAGAGTATCGAGGGC/CGACAAAGGCGGTAGA |
| *pr1A* | MAA-08701 | Subtilisin-like protease pr1A | TCTGATAGACTTTCCTCCTT/AGATTTAGTAATGCCACCT |
| *pr1C* | MAA-00492 | Subtilisin-like protease pr1C | ACAAGGCCCACGAAAAGG/AGGAAAGGAGACCACCCG |
| *chit30* | MAA-01212 | Chitinase CHIT30 | CGTACCCCGACCAAT/CCGCTCTGGGCAATG |
